# *In situ* expression of yak *DEFB127* via plasmid-mediated delivery alleviates bovine *Staphylococcus aureus*-induced mastitis through anti-inflammatory mechanisms

**DOI:** 10.3389/fvets.2026.1910975

**Published:** 2026-10-22

**Authors:** Dizhi Lu, Zening Wang, Wenying Dai, Ze Zhou, Shujun Yue, Xiangfei Zhang, Biao Li, Mingfeng Jiang, Yili Liu

**Affiliations:** 1 College of Animal Science and Veterinary Medicine, Southwest Minzu University, Chengdu, Sichuan, China; 2 Sichuan Academy of Grassland Science, Chengdu, Sichuan, China

**Keywords:** *DEFB127*, intestinal microbiota, mastitis, *Staphylococcus aureus*, yak

## Abstract

**Introduction:**

Mastitis, a disease affecting milk production and quality, poses a significant challenge to agricultural practitioners. Conventional antibiotic treatments present potential issues such as drug resistance, disruption of animal intestinal microbiota, and milk safety issues, necessitating the development of novel alternative therapies for bovine mastitis. Yak β-defensin 127 (*DEFB127*), a natural antimicrobial peptide, represents a promising candidate for alternative to antibiotics, yet remains underexplored as a therapy for mastitis.

**Results:**

In MAC-T cell, qRT-PCR analysis revealed that expression of IL-6 and IL-1β in the pLF-*DEFB127* treatment group were significantly lower than the infection group (*P* < 0.01). *In vivo*, histopathological examination showed that degree of inflammatory cell infiltration in the mammary gland tissue of the treatment group was significantly reduced compared to the infection group, accompanied by marked alleviation of alveolar dilation and epithelial cell necrosis. ELISA and qRT-PCR results demonstrated that the expression of *TNF-*α, *IL-6*, and *IL-1*β in the mammary gland tissue of mice from the pLF-*DEFB127* treatment group were significantly downregulated than those in the infection group (*P* < 0.01). Furthermore, 16S rRNA gene sequencing revealed that pLF-*DEFB127* treatment altered the structure of the mouse intestinal microbiota, increasing the relative abundance of Bacteroidia and Bacilli, while decreasing that of Gammaproteobacteria. Finally, functional prediction of the intestinal microbiota based on 16S rRNA correlation analysis indicated that yak *DEFB127* might exert anti-inflammatory effects by mechanisms involving glycosphingolipid biosynthesis and immune signaling, though this requires experimental validation.

## 1 Introduction

Mastitis is a prevalent disease in dairy cows, primarily caused by pathogenic invasion. Among these pathogens, *Staphylococcus aureus* (*S. aureus*) is one of the predominant causative agents (1). In China, the overall prevalence of *S. aureus*-induced bovine mastitis is 36.23%, affecting cow health, milk production, and quality (1). In Spain, the estimated annual economic loss per cow due to mastitis reaches up to €300 (1, 2). The yak, a semi-wild and rare livestock breed in China, produces milk that is locally regarded as “natural concentrated milk” owing to its high nutritional value. This milk serves as an important food source and a raw material for dairy processing among high-altitude populations (3, 4). However, *S. aureus* and other pathogenic invasions compromise yak health as well as milk yield and quality. Moreover, the disease is highly contagious, readily spreading within herds and causing substantial economic losses (5). Currently, *S. aureus* exhibits increasing resistance to commonly used antibiotics, including β-lactams, macrolides, and tetracyclines. Moreover, antibiotic treatment disrupts the gastrointestinal microbiota of cattle, while drug residues in dairy products pose risks to animal health and food safety (6, 7). The overuse of antibiotics in mastitis treatment has created a vicious cycle of escalating resistance, disruption of the bovine gastrointestinal microbiota, and food safety threats from antibiotic residues in dairy products. Consequently, the development of effective alternative therapies with minimal adverse effects has become an urgent priority in veterinary medicine (5, 6).

To address these challenges, recent studies have explored alternative strategies targeting mitochondrial dysfunction, which is characterized by enhanced mitochondrial metabolism in mammary tissue during mastitis. For instance, Forsythiaside A has been shown to alleviate mastitis by activating the PINK1/Parkin pathway and accelerating mitophagy (8). Additionally, quercetin, a natural compound, has been shown to alleviate bovine mastitis by modulating histone acetylation and DNA methylation, as well as regulating signaling pathways including NF-κB and the NLRP3 inflammasome (9). Antimicrobial peptides (AMPs) represent another promising antibiotic alternative. These low-molecular-weight peptides are widely distributed in nature and serve as key effectors of innate immunity. Moreover, AMPs exhibit direct bactericidal and immunomodulatory activities, coupled with broad-spectrum efficacy, reduced resistance potential, and favorable safety profiles (10).

Notably, β-defensins, as mammalian AMPs, display potent broad-spectrum antimicrobial activity (10). β-defensins are known to function via dual mechanisms: direct antimicrobial activity and immunomodulation. While the antimicrobial activity of *DEFB127* has been inferred from its structural features, the present study focuses on evaluating its anti-inflammatory potential in mastitis treatment (11). Its antimicrobial activity is mediated by the cationic nature of the molecule, which facilitates electrostatic interactions with negatively charged bacterial membranes. This interaction promotes peptide insertion into the lipid bilayer, leading to pore formation, membrane depolarization, and ultimately cell lysis (12, 13). In terms of immunomodulation, β-defensins can recruit monocytes, macrophages, and immature dendritic cells via chemotaxis and induce mast cell activation and degranulation. The latter releases inflammatory mediators such as histamine and prostaglandin D2, which subsequently recruit neutrophils. These neutrophils then degranulate and release endogenous defensins, thereby forming a defensin–immune cell positive feedback loop that amplifies the host immune response (12, 13). Our previous studies have investigated various β-defensins. For example, yak *DEFB123* was shown to alleviate Klebsiella pneumoniae-induced lung injury via the MAPK signaling pathway (14). Yak *DEFB124* alleviate *S. aureus* infection-induced intestinal injury (15); Yak *DEFB116* plays an important role in combating *S. aureus* and maintaining intestinal homeostasis (16). Research on *DEFB127*, a member of the β-defensin family, remains limited, particularly regarding its application in bovine mastitis. Among β-defensins, *DEFB127* represents a promising yet underexplored candidate for antibiotic replacement therapy.

The central scientific question of this study is whether plasmid-mediated *in situ* expression of yak *DEFB127* can serve as a safe and effective antibiotic alternative for treating *S. aureus*-induced mastitis. Conventional antibiotic therapy, while effective against pathogens, inevitably disrupts the host intestinal microbiota and contributes to resistance development. In contrast, antimicrobial peptides such as *DEFB127* offer a dual advantage: direct bactericidal activity and immunomodulation, with potentially fewer off-target effects on commensal microbiota. We hypothesized that breast-specific plasmid delivery of yak *DEFB127* would (i) effectively alleviate mammary inflammation *in vitro* and *in vivo*, and (ii) exert less perturbation on the intestinal microbiota compared to conventional antibiotic treatment. To test this hypothesis, we first characterized the molecular properties of yak *DEFB127* through bioinformatics analysis. We then evaluated its therapeutic efficacy in a bovine mammary epithelial cell (MAC-T) model and an ICR mouse mastitis model. Finally, we assessed the impact of pLF*-DEFB127* treatment on the intestinal microbiota composition relative to lincomycin treatment, as a critical measure of its safety profile as an antibiotic alternative. This study aims to explore novel plasmid-based delivery strategies for mastitis treatment using β-defensin-derived peptides with known antimicrobial properties, focusing herein on their anti-inflammatory effects, microecological regulatory effects on the intestinal microbiota, and the safety advantages of minimal microbiota perturbation compared to conventional antibiotics.

## 2 Methods

### 2.1 Experimental material

#### 2.1.1 Source of samples

The plasmid pLF-EGFP was a mammary gland-specific plasmid obtained through preliminary screening (4). The *S. aureus* strain used in this study was provided by Professor Yongjiang Mao (Yangzhou University, China). The MAC-T cell line was obtained from Professor Yanying Zhao (Southwest Minzu University, China). Specific pathogen-free (SPF) ICR mice (8 weeks old, female) were purchased from Chengdu Dashuo Laboratory Animal Co., Ltd. (Chengdu, China).

#### 2.1.2 Main reagents

The following reagents were used in this study: genomic DNA extraction kit and endotoxin-free plasmid DNA purification kit (TianGen Bio-Chem Technology, Beijing, China); PrimeSTAR^®^ Max DNA Polymerase, Kpn I, Xba I, restriction enzymes (Thermo Fisher Scientific, Beijing, China); *Escherichia* coli DH5α competent cells (Takara, Beijing, China); enzyme-linked immunosorbent assay (ELISA) kit (Bioswamp, Wuhan, China); DMEM/F12, Opti-MEM, and DMEM high glucose medium (Gibco Life Sciences, Waltham, MA, USA); penicillin–streptomycin (Biosharp, Beijing, China); fetal bovine serum (Biological Industries, Beijing, China); TB Green^®^ Premix Ex Taq, and PrimeScript™ FAST RT reagent kit with gDNA Eraser (Takara, Beijing, China); and Lipofectamine 2000 (Thermo Fisher Scientific, Beijing, China).

### 2.2 Gene amplification and bioinformatics analysis

Total RNA was extracted from yak spleen tissue and reverse transcribed into complementary DNA (cDNA). Gene-specific primers were designed based on the yak *DEFB127* cDNA sequence (GenBank accession number: XM_005887947.1), as listed in Table 1. Yak spleen cDNA was used as a template to amplify the coding region of the yak *DEFB127* gene by PCR. The 20 μL reaction mixture contained 10 μL PrimeSTAR^®^ Max DNA Polymerase, 0.4 μL each of forward and reverse primers (10 μM), 2 μL template cDNA, and nuclease-free water to a final volume of 20 μL. Thermal cycling conditions were as follows: initial denaturation at 95 °C for 30 s; followed by 40 cycles of denaturation at 98 °C for 10 s, annealing at 55 °C for 15 s, and extension at 68 °C for 30 s. Amplification products were verified by agarose gel electrophoresis. Sequence homology analysis and phylogenetic tree construction were performed using MegAlign Pro (DNASTAR, Madison, WI, USA) and MEGA 7.0 (https://www.megasoftware.net), respectively. The physicochemical properties of yak *DEFB127* were analyzed using the ProtParam tool (https://web.expasy.org/protparam/), and the tertiary structure was predicted by homology modeling using the SWISS-MODEL web server (17) (https://swissmodel.expasy.org/~).

**Table 1:** RT-qPCR primer sequences for *GAPFH, TNF*-α, *IL*-*6*, and *IL*-*1*β in cells and mice.

Primer sequence		
Genes	Length	Primer sequence (5′-3′)
*DEFB127*-F	132 bp	GGGGTACCATGAGGCTCCTCCTGATCATTG
*DEFB127*-R		GCTCTAGATTATGTAGGCTTTCTGTGGGAGT
*GAPDH*-F-Cell	208bp	TGTTGTGGATCTGACCTGCC
*GAPDH*-R-Cell		AAGTCGCAGGAGACAACCTG
*GAPDH*-F-Mice	130bp	AGGTCGGTGTGAACGGATTT
*GAPDH*-R-Mice		GGGGTCGTTGATGGCAACA
*TNF-α*-F-Cell	126bp	GTTCTCCCCATGACACCACCTG
*TNF-α*-R-Cell		GGGAGAAGAGAGTCAGACAGGC
*IL-6*-F-Cell	134bp	ACAAGCGCCTTCACTCCATT
*IL-6*-R-Cell		AGAAGTAGTCTGCCTGGGGT
*IL-1β*-F-Cell	140bp	ATGGCAACCGTACCTGAACC
*IL-1β*-R-Cell		CCATCTCCCATGGAACCGAG
*TNF-α*-F-Mice	126bp	CCACCACGCTCTTCTGTCTA
*TNF-α*-R-Mice		CCACTTGGTGGTTTGTGAGTG
*IL-6*-F-Mice	138bp	GGTCTTCTGGAGTACCATAGC
*IL-6*-R-Mice		GTGACTCCAGCTTATCTCTTGGT
*IL-1β*-F-Mice	136bp	TGCCACCTTTTGACAGTGATG
*IL-1β*-R-Mice		ATGTGCTGCTGCGAGATTTG

### 2.3 Construction of the pLF-*DEFB127* plasmid vector

The mammary-specific plasmid pLF-EGFP (containing the lactotransferrin, LF, promoter) and the yak *DEFB127* gene fragment were digested with Kpn I and Xba I. The digested fragments were ligated with T4 DNA ligase at 16 °C for 16 h to construct the recombinant plasmid pLF-*DEFB127*, which was then transformed into Escherichia coli DH5α competent cells. Colony PCR was performed to screen positive clones, and the amplified products were analyzed by agarose gel electrophoresis. Positive clones were sequenced by Sangon Biotech (Shanghai, China), and the sequences were verified by BLAST analysis (4, 15).

### 2.4 *S. aureus* mastitis cell model experiment

#### 2.4.1 Plasmid transfection

MAC-T cells were thawed and cultured in complete medium. Upon reaching approximately 90% confluence, cells were trypsinized and seeded into 12-well plates. The pLF-*DEFB127* plasmid was purified using an endotoxin-free plasmid kit and transfected into MAC-T cells using Lipofectamine 2000 (1 μg per well). At 48 h post-transfection, total RNA was extracted from transfected and control cells, reverse transcribed into cDNA using the PrimeScript™ FAST RT Kit with gDNA Eraser, and analyzed by RT-qPCR to assess plasmid overexpression efficiency (17).

#### 2.4.2 Infection cells model construction

The *S. aureus* strain was thawed and revived, then inoculated into LB broth. Following incubation at 37 °C to the exponential growth phase, the bacterial concentration was adjusted to 1 × 10^8^ CFU/mL by turbidimetry, and bacterial cells were harvested by centrifugation for subsequent use. MAC-T cells were seeded at a density of 1 × 10^6^ cells/well in 6-well plates and divided into four treatment groups (17). Were revived from frozen stocks and inoculated into LB broth. After incubation at 37 °C to the log phase, bacterial density was adjusted to 1 × 10^8^CFU/mL by turbidimetry, and bacteria were harvested by centrifugation for subsequent use. MAC-T cells were seeded at a density of 1 × 10^6^ cells/well in 6-well plates and divided into four treatment groups (*n* = 3 per group): (i) normal control (NC), cells cultured in complete medium; (ii) *S. aureus* infection, cells infected with *S. aureus* at a multiplicity of infection (MOI) of 10 for 4 h; (iii) positive control (PC), cells infected as in (ii), washed three times with PBS, and cultured in complete medium containing 10 μg/mL penicillin-streptomycin for 24 h; and (iv) pLF-*DEFB127*, cells transfected with pLF-*DEFB127* for 48 h prior to *S. aureus* infection (MOI = 10) for 4 h.

### 2.5 ICR mouse mastitis model

#### 2.5.1 Infection mouse model construction

Female ICR mice (8 weeks old, SPF) were housed under controlled conditions (22 ± 2 °C, 12 h light/dark cycle) with free access to standard chow and water. Lactating mice at 7 days postpartum were randomly assigned to four groups (*n* = 6 per group) with comparable body weights. The fourth inguinal mammary glands were challenged bilaterally as follows: (1) NC group: 50 μL sterile saline injected per gland, followed by 20 μL sterile saline at 24 h post-injection; (2) *S. aureus* group: 50 μL *S. aureus* suspension (1 × 10^7^ CFU/mL) per gland; (3) PC group: 50 μL *S. aureus* suspension (1 × 10^7^ CFU/mL) per gland, followed by intramammary administration of 20 μL lincomycin (1.5 g/mL) at 24 h post-infection; (4) pLF-*DEFB127* group: 50 μL *S. aureus* suspension (1 × 10^7^ CFU/mL) per gland, followed by 20 μL pLF-*DEFB127*/Pluronic L64 mixture (1:1, v/v; plasmid concentration: 1000 ng/μL; final L64 concentration: 0.1%) at 24 h post-infection. All injections were performed using U-40 insulin syringes. The aforementioned concentrations were all obtained from the research group's preliminary studies (17).

#### 2.5.2 Anatomical observation and specimen collection of mice

At 48 h post-treatment (72 h post-infection), mice were euthanized via intraperitoneal administration of an overdose of anesthetic (ketamine 180 mg/kg and xylazine 20 mg/kg body weight, i.p.), followed by cervical dislocation as a secondary confirmatory method (18). Body weights were measured at three time points: prior to the first injection, prior to the second injection (24 h post-infection), and before sacrifice. General clinical condition and teat skin integrity were assessed prior to euthanasia. Following laparotomy, macroscopic examination of the fourth mammary glands was performed to evaluate color, size, consistency, hemorrhagic lesions, and periglandular tissue changes. Specimens of the fourth mammary glands and intestinal luminal contents were collected, briefly rinsed in ice-cold PBS (mammary glands only), flash-frozen in liquid nitrogen, and preserved at −80 °C until further analysis.

#### 2.5.3 Pathological observation of mouse mammary gland tissue

Mammary gland tissues were fixed in 4% paraformaldehyde for 24 h, then dehydrated, embedded, sectioned, and deparaffinized. Sections were stained with hematoxylin for 10–20 min, rinsed in tap water for 1–3 min, differentiated in 1% hydrochloric acid–ethanol for 5–10 s, and rinsed again in tap water for 1–3 min. Tissues were then immersed in warm water (50 °C) or a weakly alkaline solution until blue coloration appeared, rinsed in tap water for 1–3 min, dehydrated in 85% ethanol for 3–5 min, and stained with eosin for 3–5 min. After rinsing for 3–5 s, sections were subjected to gradient ethanol dehydration, xylene clearing, and mounting with neutral balsam. Finally, sections were examined under a light microscope.

#### 2.5.4 Detection of inflammatory factors in mouse mammary gland tissue by ELISA

Tissue supernatants were prepared according to the ELISA kit manufacturer's instructions, and *TNF-*α, *IL-6*, and *IL-1*β concentrations were quantified. Concentrations were calculated from absorbance values using the formula provided in the kit manual.

#### 2.5.5 Real-time fluorescent quantitative pcr of inflammatory factors in mouse mammary gland

The expression levels of *DEFB127* and inflammatory cytokine genes (*TNF-*α, *IL-6*, and *IL-1*β) in MAC-T cells were determined by RT-qPCR using *GAPDH* as the internal control, to evaluate *DEFB127* overexpression and its anti-inflammatory potential. Primers were designed based on sequences retrieved from NCBI and synthesized by Sangon Biotech (Shanghai, China) (Table 1). RT-qPCR was performed using a real-time PCR system, and relative gene expression was calculated using the 2^−Δ*ΔCt*^ method.

The following RT-qPCR reaction system was established according to the instructions for the TB GreenTM Premix Ex TaqTM II kit. The RT-qPCR reaction system volume is 20 μL: 2 × TB Green™ Premix Ex Taq™ II (10 μL), forward and reverse primers (10 μmol/L) at 0.8 μL each, MAC-T cell cDNA (6.4 μL) and ddH_2_O (2 μL). RT-qPCR procedure: 95 °C pre-denaturation for 3 min; 95 °C denaturation for 10 s, 60 °C annealing for 15 s, 72 °C extension for 30 s, repeated for 40 cycles. The properly labeled centrifuge tubes were placed in the high-throughput real-time fluorescence quantitative instrument LightCycler 96 for testing. After setting the program, the operation was initiated. The relative expression levels of inflammatory factors were calculated using the 2^−Δ*ΔCt*^ method.

#### 2.5.6 Assessment of intestinal microbiota as a safety indicator of antibiotic alternative therapy

To assess the microbiota-sparing effect of pLF-*DEFB127* relative to conventional antibiotics, fecal microbiota composition was analyzed across all experimental groups. DNA was extracted from fecal samples, and the V3-V4 region of the 16S rRNA gene was amplified and quantified using a Qubit 2.0 fluorometer. Paired-end sequencing (PE250) was performed on the NovaSeq 6000 (Illumina). Raw reads were quality-filtered and analyzed using QIIME 2. All molecular procedures were conducted by Aochuang Biotechnology (Chengdu, China).

### 2.6 Statistical analysis

SPSS Statistics 26 was used for analysis of variance (ANOVA), with multiple comparisons performed using the LSD method. An asterisk (^*^) indicates a statistically significant difference (*P* < 0.05), while a double asterisk (^**^) denotes an extremely significant difference (*P* < 0.01).

## 3 Results

### 3.1 Bioinformatics analysis of yak *DEFB127*

#### 3.1.1 Sequence homology and phylogenetic tree analysis

The yak *DEFB127 sequence* shared the highest identity (99.7%) with *Bos indicus* × *Bos taurus* (Figure 1A). Its CDS was highly conserved and showed high homology with *Bubalus kerabau, Bos taurus*, and *Bos indicus* × *Bos taurus* (Figure 1B). Phylogenetic analysis revealed that yak DEFB127 protein clustered with these three species, with *Bubalus kerabau* showing the second-highest sequence identity.

**Figure 1:**
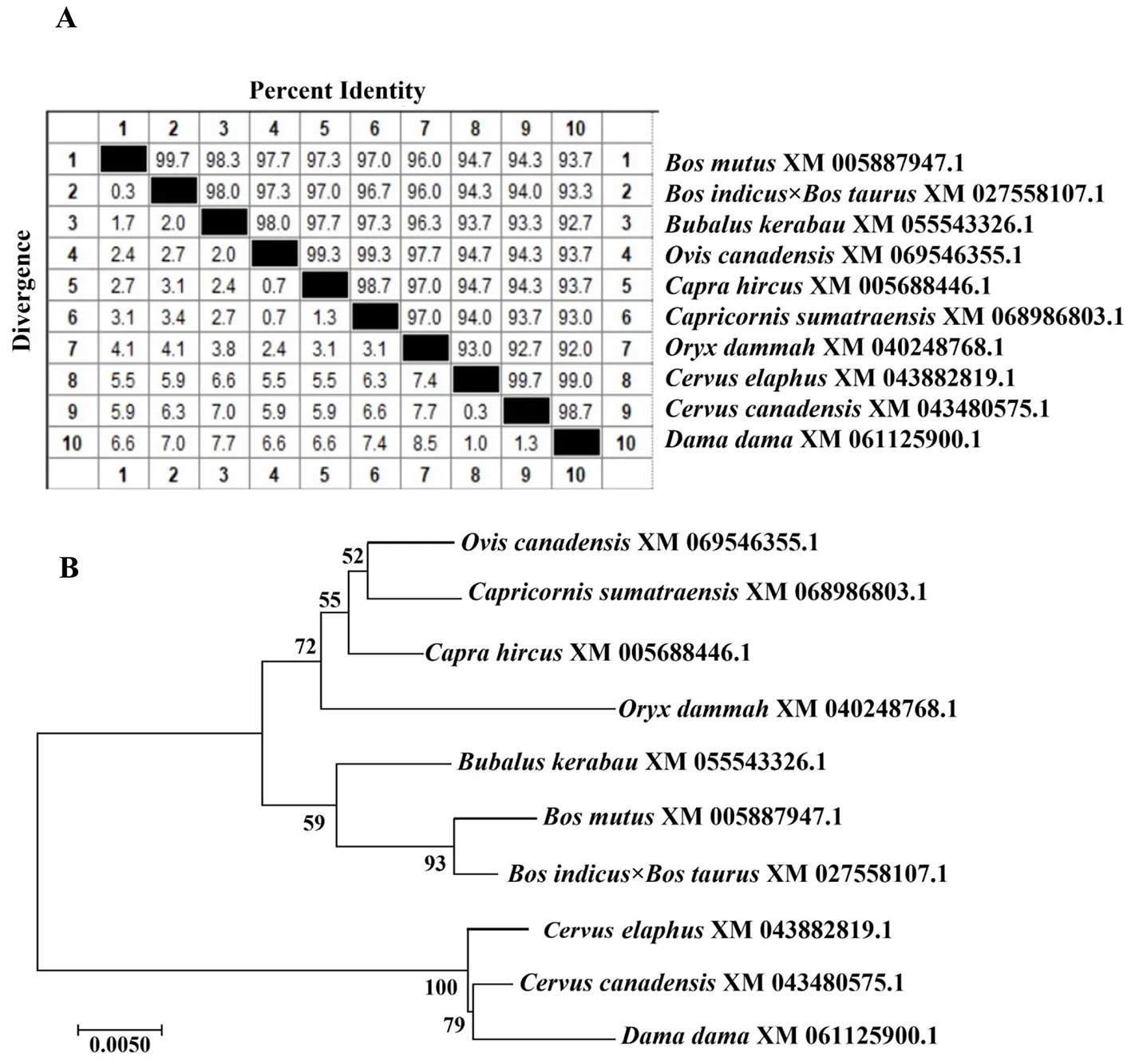
Sequence analysis of the yak *DEFB127* gene. **(A)** Homology analysis of yak *DEFB127*. **(B)** Phylogenetic tree of yak *DEFB127*.

#### 3.1.2 Primary structure and physicochemical properties

To evaluate the physicochemical properties associated with antimicrobial activity, the primary structure of yak *DEFB127* was analyzed. Yak *DEFB127* has a molecular formula of C_525_H_862_N_156_O_142_S_7_ and contains 18 positively charged and 9 negatively charged residues (Figure 2A). The instability index (40.52) predicts an unstable protein with a 30 h half-life, whereas the GRAVY value (−0.392) indicates hydrophilicity. The 300-bp ORF encodes 99 amino acids, with hydrophobic residues comprising 33%, a proportion exceeding the 30% threshold typical of AMPs (Figure 2B).

**Figure 2:**
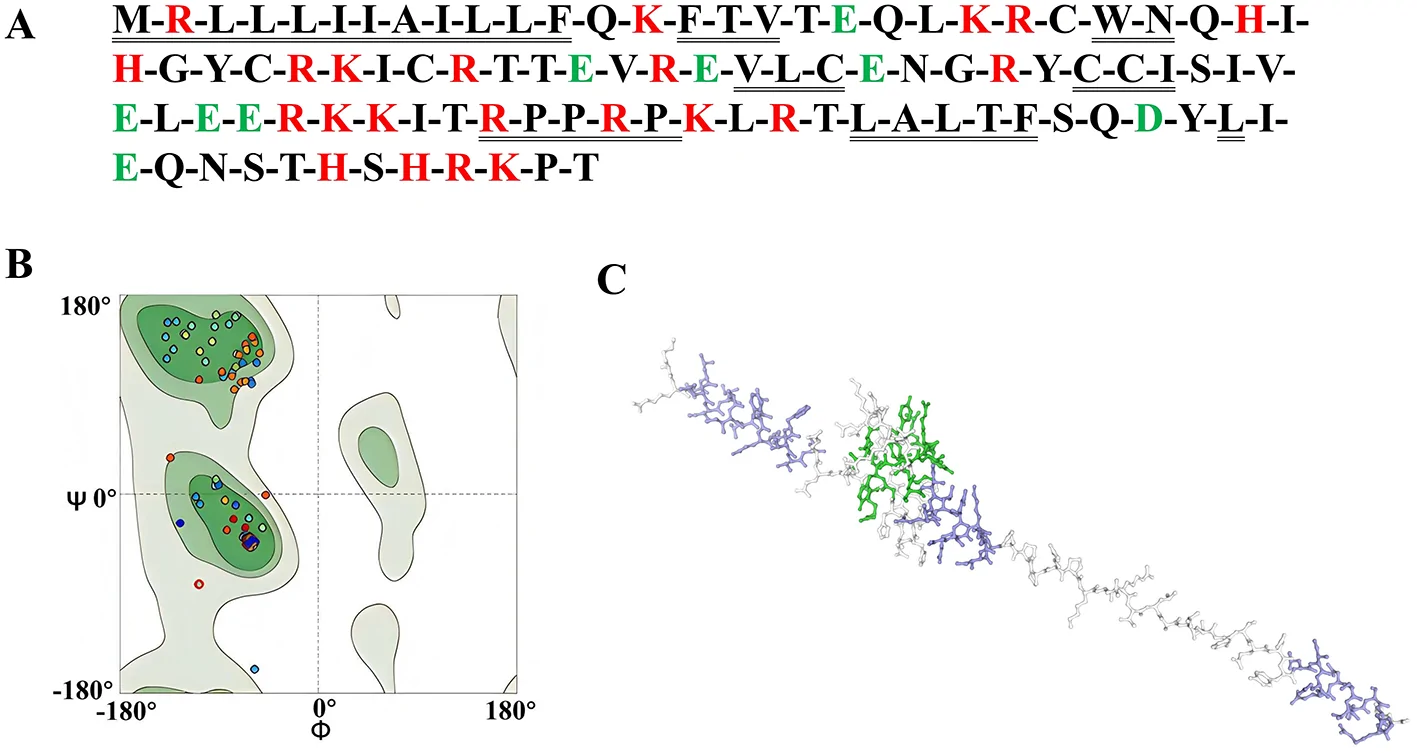
Structural analysis of yak DEFB127 protein. **(A)** DEFB127 protein sequence; red letters represent positively charged amino acids; green letters represent negatively charged amino acids; double solid underlines indicate hydrophobic residues. **(B)** Ramachandran plot of amino acid residues in the yak DEFB127 protein; dark green, light green, and white regions represent the most favored, allowed, and disallowed regions, respectively. **(C)** Tertiary structure of the yak DEFB127 protein.

#### 3.1.3 Protein tertiary structure prediction

To assess the structural feasibility of yak *DEFB127*, we predicted its tertiary structure and evaluated stereochemical quality. The Ramachandran plot exhibited green, light green, and white regions corresponding to the most preferred, additionally allowed, and disallowed regions, respectively (Figure 2B). The predicted three-dimensional structure revealed α-helical predominance (Figure 2C). Like most well-folded proteins, the majority of *DEFB127* residues were located in the most preferred and additionally allowed regions, consistent with stereochemical principles underlying protein stability. These findings collectively supported the hypothesis that yak *DEFB127* is a structurally validated β-defensin amenable to functional evaluation in mastitis models.

### 3.2 Analysis of the anti-inflammatory effect of *DEFB127* in MAC-T cells

#### 3.2.1 Transfection detection of pLF-*DEFB127* plasmid

To establish a cell-based model for *DEFB127* functional evaluation, the pLF-*DEFB127* plasmid was constructed and its expression in MAC-T cells was confirmed. Agarose gel electrophoresis revealed a 300-bp band, consistent with the expected yak *DEFB127* amplicon size (Figure 3A). Plasmid sequence verification is shown in Figure 3B. RT-qPCR analysis demonstrated significantly elevated *DEFB127* expression in transfected cells compared with the control group (Figure 3C). Collectively, these findings confirmed successful plasmid construction and transfection efficiency, establishing a foundation for subsequent anti-inflammatory functional assays.

**Figure 3:**
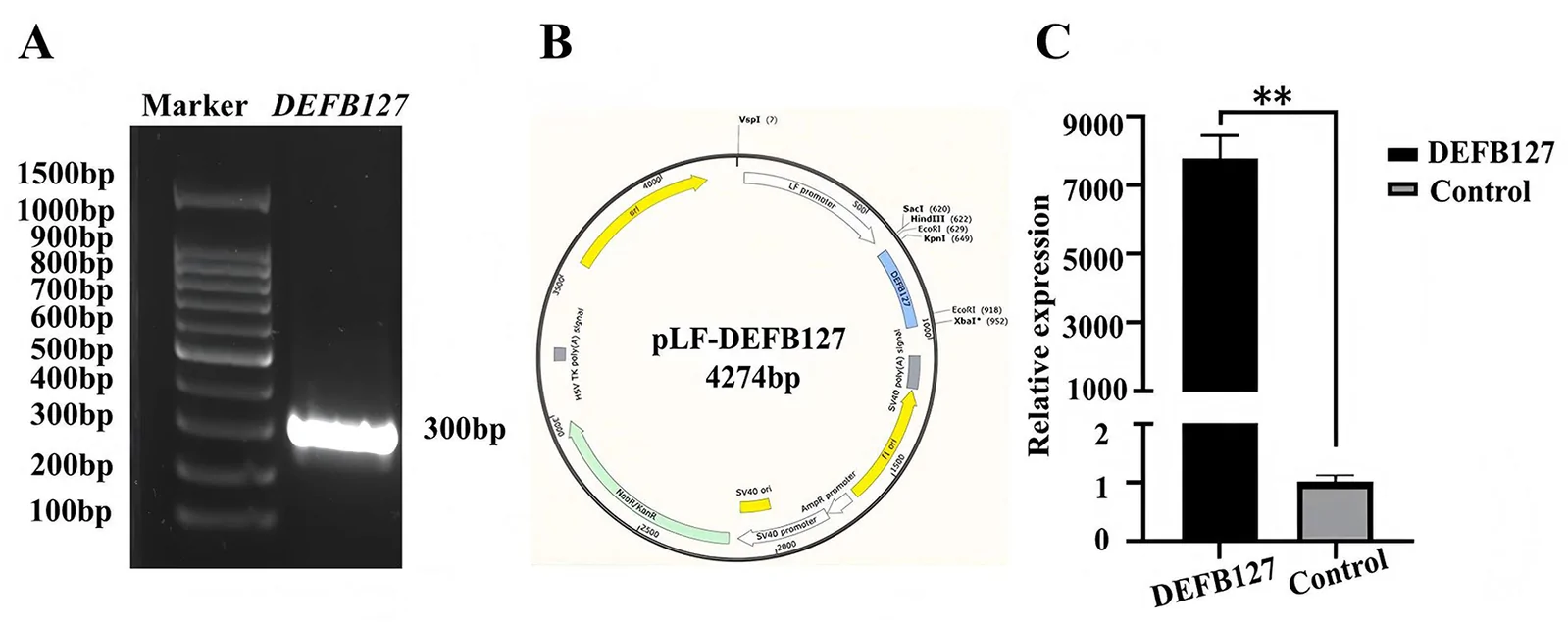
*DEFB127* gene validation. **(A)** PCR amplification of the *DEFB127* gene. **(B)** Plasmid map of recombinant pLF–*DEFB127* plasmid. **(C)** RT qPCR detection of *DEFB127* expression after pLF–*DEFB127* plasmid transfection into MAC–T cells. *P* < 0.01 indicates a highly significant difference (**).

#### 3.2.2 Anti-inflammatory effect of *DEFB127* in S. aureus-infected MAC-T cells

To evaluate whether *DEFB127* expression attenuates *S. aureus*-induced inflammation in mammary epithelial cells, we established an *in vitro* mastitis model by infecting MAC-T cells with *S. aureus* (MOI = 10) and assessed inflammatory cytokine expression by RT-qPCR. It revealed that *IL-6* and *IL-1*β expression was significantly higher in the *S. aureus* group than in the NC group (*P* < 0.01). *TNF-*α, *IL-6*, and *IL-1*β expression was highest in the *S. aureus* group (Figure 4), indicating that the cell model of *S. aureus* infection was successfully established. Following *DEFB127* expression, inflammatory factor levels in transfected cells were significantly lower than those in the *S. aureus* group (*P* < 0.01). Analysis of *IL-6* and *IL-1*β expression showed that their levels in the pLF-*DEFB127* group were significantly reduced compared with those in the *S. aureus* group.

**Figure 4:**
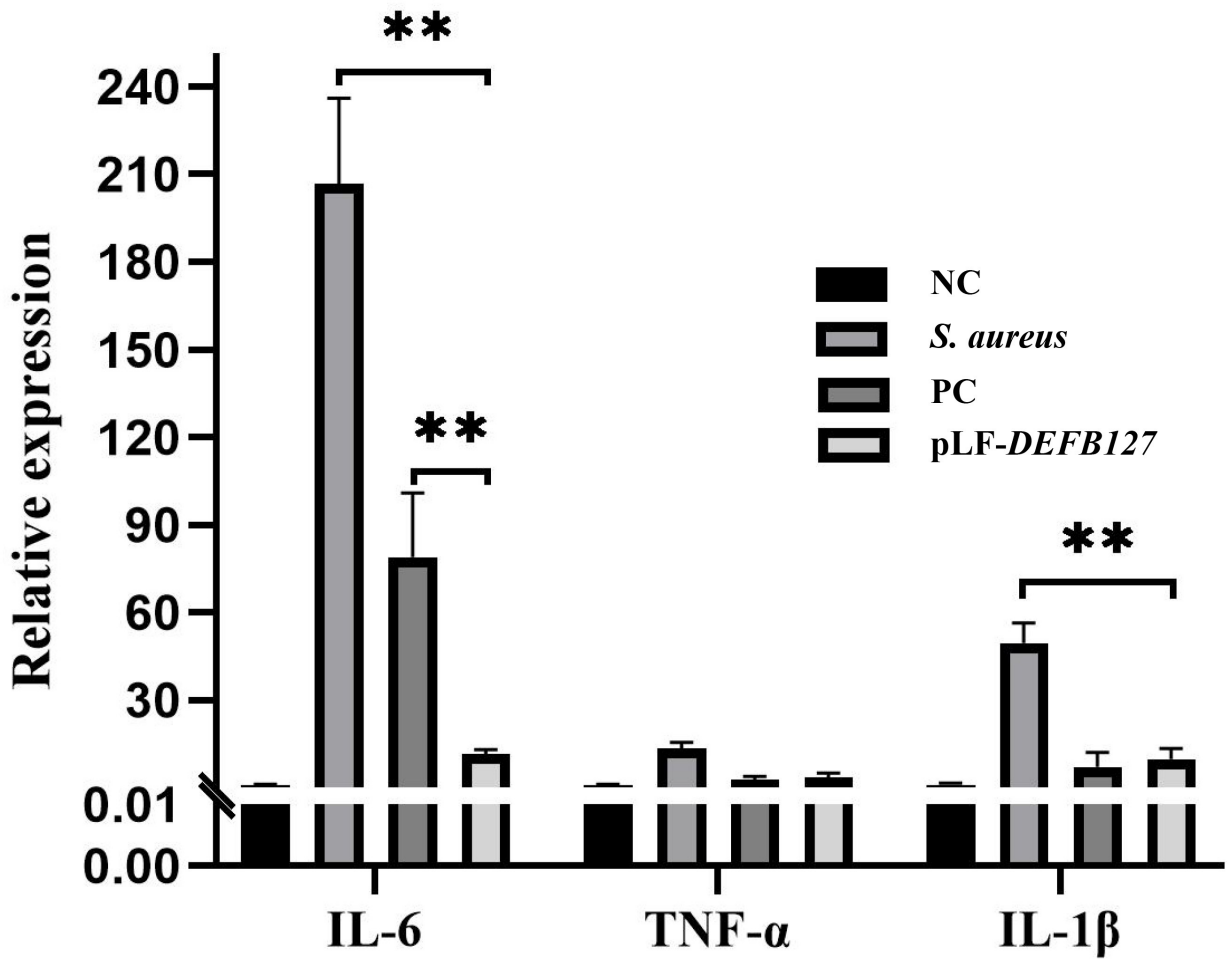
RT-qPCR detection of inflammatory–factor expression levels in MAC–T cells under different treatments. *P < 0.05, **P < 0.01.

### 3.3 Analysis of the therapeutic efficacy of pLF-*DEFB127* in a mouse mastitis model

#### 3.3.1 Body weight changes of mice

To evaluate the systemic impact of *S. aureus* infection and the therapeutic effects of *DEFB127* treatment *in vivo*, we first monitored body weight changes as an indicator of overall health status. Mice showed no significant differences in initial body weight among all groups, but body weight changed significantly in the treated groups after treatment (Figure 5A). Body weight decreased in all groups except the NC group, with the *S. aureus* group showing the greatest decrease, followed by the pLF-*DEFB127* group and the PC group (Figure 5B).

**Figure 5:**
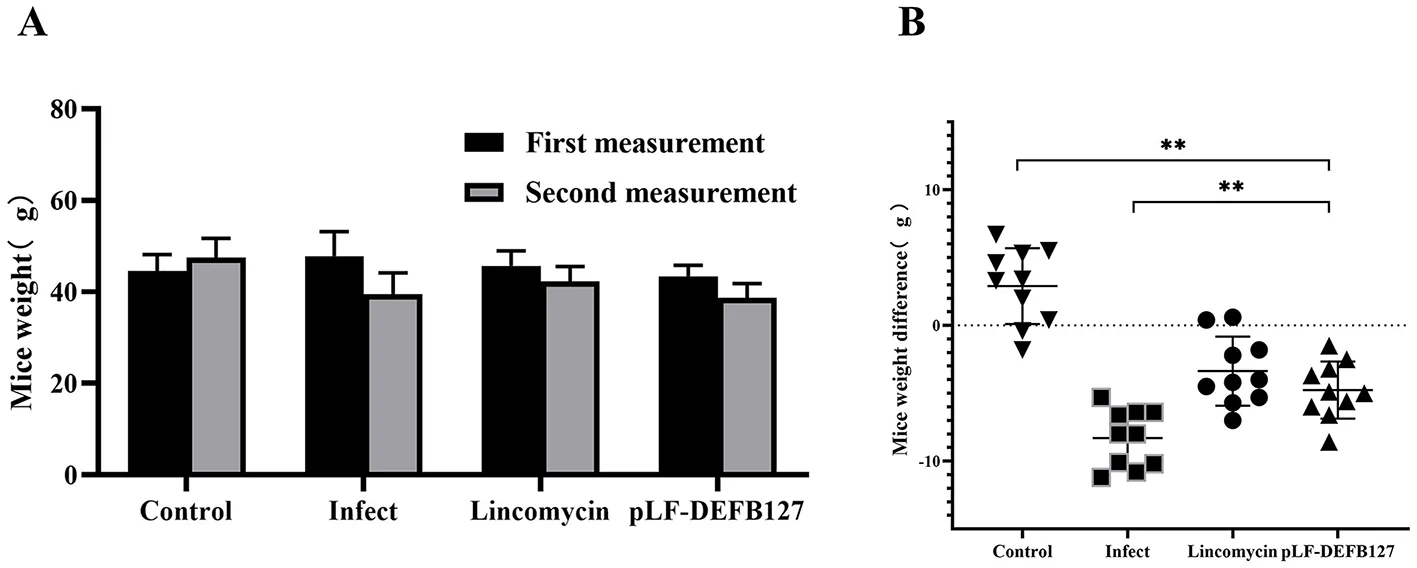
Change in body weight of mice. **(A)** Body weights of mice before the first injection and before the second injection. **(B)** Differences in body weight among mice in each group. *P < 0.05, **P < 0.01.

#### 3.3.2 Anatomical observations of mammary gland tissue

To visually assess gross pathological changes in mammary tissue following *S. aureus* infection and *DEFB127* treatment, gross pathological examination of the fourth pair of mammary glands was performed. Representative images of mammary glands from each group are shown in Figure 6A. In the NC group, mammary glands were well-developed and uniform in shape, with a cream-colored appearance. In the *S. aureus* group, obvious hemorrhagic spots were visible, with varying degrees of swelling and erosion; the overall color was dark red, indicating a severe inflammatory response. In the PC group, minor hemorrhagic spots were observed, with a color slightly darker than that of the NC group. In the pLF-*DEFB127* group, local hemorrhagic spots were present, with milk-like secretions visible, and the color was slightly redder than that of the NC group.

**Figure 6:**
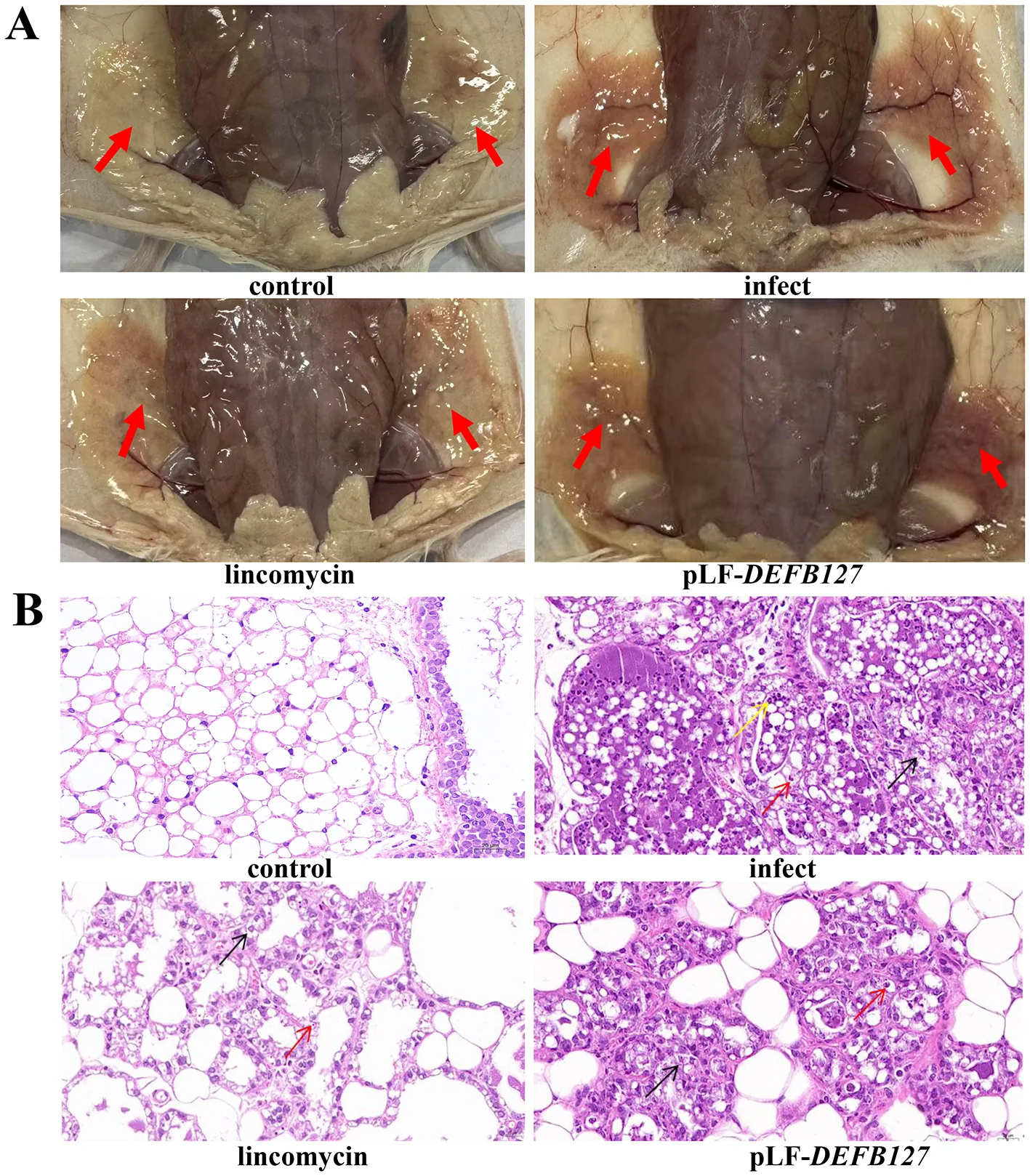
Pathological anatomy of mouse mammary glands. **(A)** Anatomical observations of mammary glands in each group of mice, with red arrows indicating mammary glands. **(B)** Pathological observations of mammary glands in each group of mice. Red arrows indicate epithelial cell necrosis, black arrows indicate epithelial cell degeneration, and yellow arrows indicate secretions and inflammatory cells.

#### 3.3.3 Histopathological changes

To evaluate the protective effects of *DEFB127* on mammary tissue architecture and cellular integrity, H&E staining was performed. Histopathological examination revealed no significant lesions in the NC group. In the *S. aureus* group, acinar dilation, epithelial cell degeneration (black arrows), occasional epithelial cell necrosis (red arrows), and luminal accumulation of secretions and inflammatory cells (yellow arrows) were observed, indicating a severe inflammatory response. In the PC and pLF-*DEFB127* groups, acinar dilation, epithelial cell degeneration, and occasional necrosis were observed (Figure 6B). However, compared with the *S. aureus* group, the pLF-*DEFB127* group exhibited reduced epithelial cell necrosis and no significant inflammatory cell infiltration.

#### 3.3.4 Detection of inflammatory factors in mammary tissue

To quantitatively assess *DEFB127* anti-inflammatory efficacy, we measured *TNF-*α, *IL-6*, and *IL-1*β expression in mammary tissues by RT-qPCR and ELISA. RT-qPCR showed significantly lower mRNA levels of all three cytokines in the pLF-*DEFB127* group compared with the *S. aureus* group (*P* < 0.01; Figure 7A). ELISA demonstrated corresponding reductions in protein levels, with IL-1β showing the most significant decrease (*P* < 0.01), followed by TNF-α (*P* < 0.05), and IL-6 showing the smallest reduction (Figure 7B).

**Figure 7:**
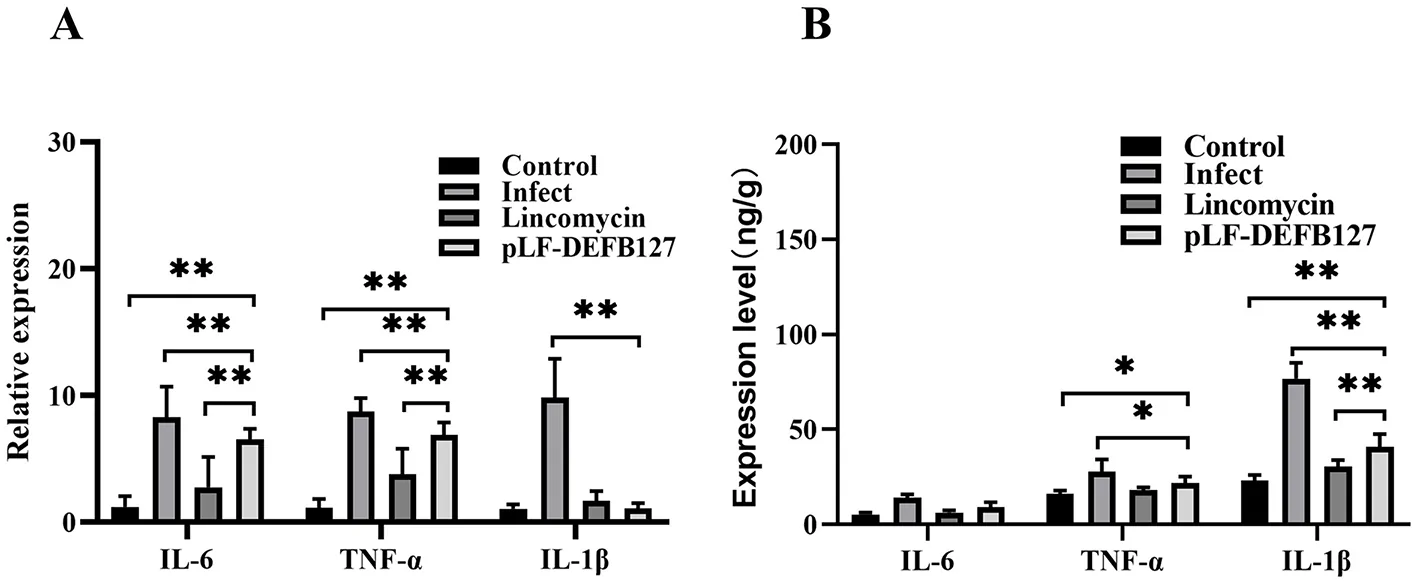
Changes in the expression of inflammatory factors. **(A)** RT-qPCR detection of inflammatory factor expression in mouse mammary inflammation treatment (*n* = 6). **(B)** Protein content of inflammatory cytokines in mouse mammary gland tissue supernatant. *P < 0.05, **P < 0.01.

### 3.4 pLF-*DEFB127* treatment is associated with less perturbation on intestinal microbiota compared to antibiotic therapy

To evaluate the microbiota-sparing potential of pLF-*DEFB127* relative to conventional antibiotics, intestinal microbiota composition was analyzed by 16S rRNA gene sequencing. As shown in Figure 8, a Venn diagram illustrated shared and unique OTUs across the four groups. Total OTU numbers for the NC, *S. aureus*, PC, and pLF-*DEFB127* groups were 827, 869, 860, and 797, respectively, with unique OTUs numbering 376, 384, 667, and 374, respectively. Shared OTUs across all groups totaled 116. Notably, the PC group exhibited substantially more unique OTUs (667) than the other groups, suggesting that lincomycin treatment introduced distinct microbial taxa absent under other conditions. At the class level, the pLF-*DEFB127* group showed increased Bacteroidetes and decreased Gammaproteobacteria relative to the NC and *S. aureus* groups, and reduced Clostridia compared with the NC group (Figure 9). Rarefaction curves reached a plateau across all groups, indicating adequate sequencing depth (Figure 10). Principal Component Analysis (PCA) based on Bray-Curtis dissimilarity revealed that the NC, *S. aureus*, and pLF-*DEFB127* groups clustered together, whereas the PC group formed a distinct cluster (Figure 11).

**Figure 8:**
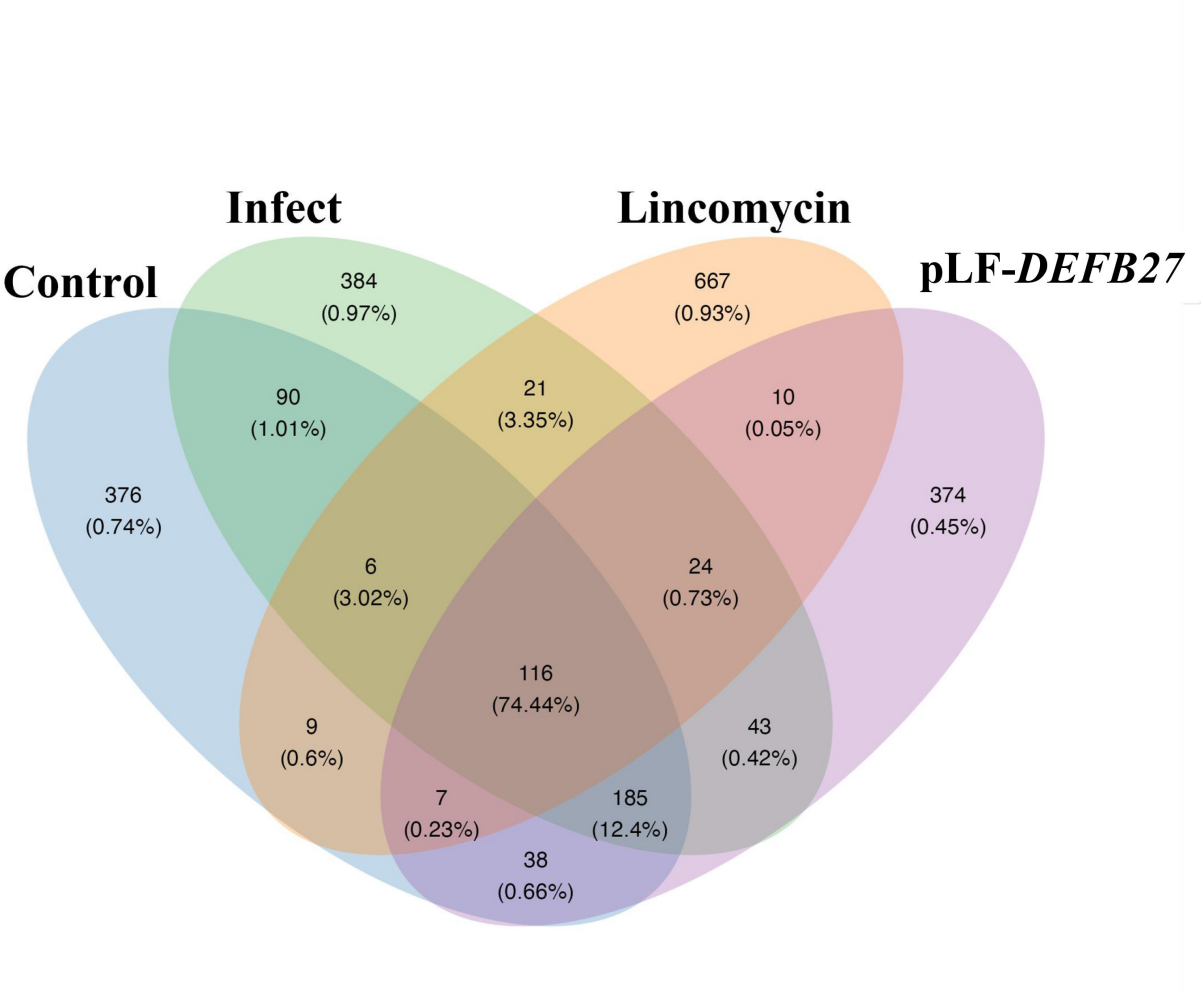
Venn diagram of mouse gut microbiota composition and diversity across experimental groups (*n* = 6 per group). Numbers in each sector indicate the count of OTUs exclusively present or shared between groups.

**Figure 9:**
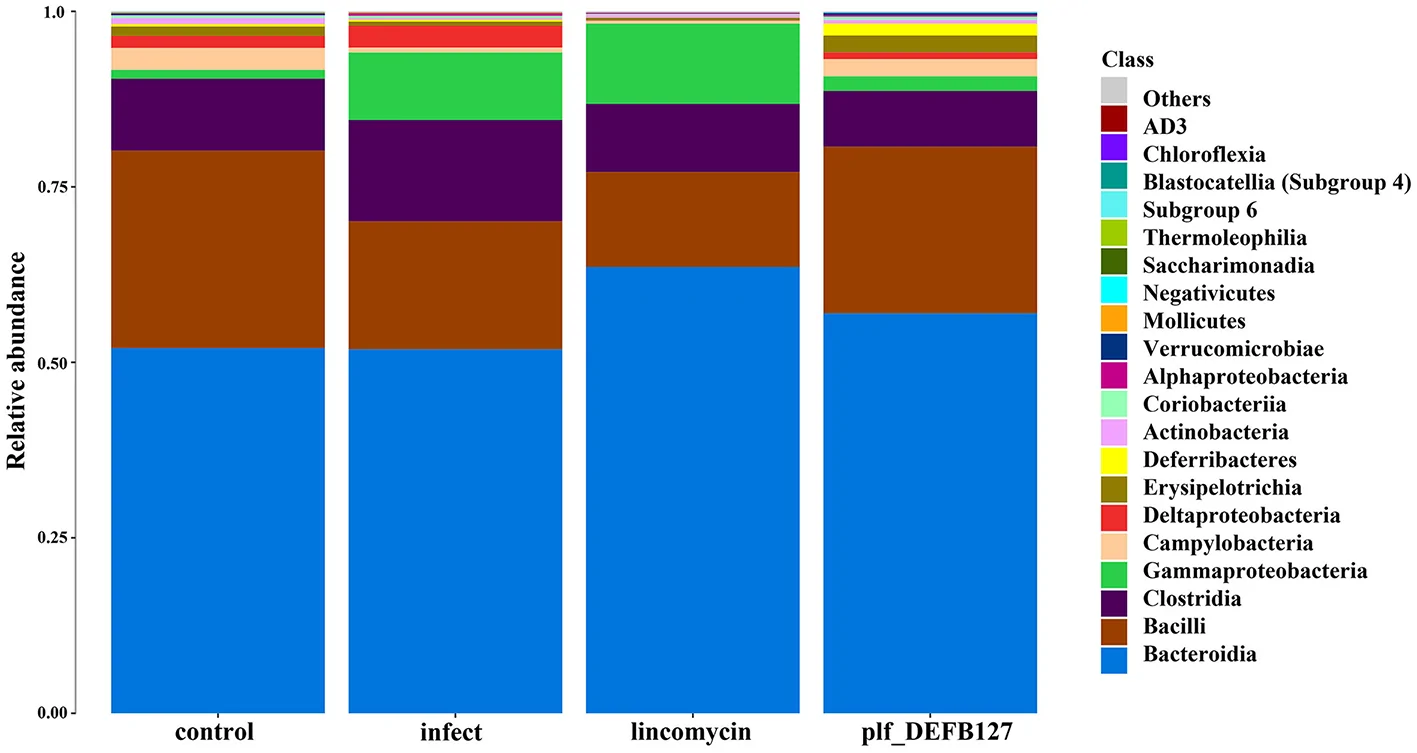
Relative abundance of intestinal microbiota at the class level across treatment groups.

**Figure 10:**
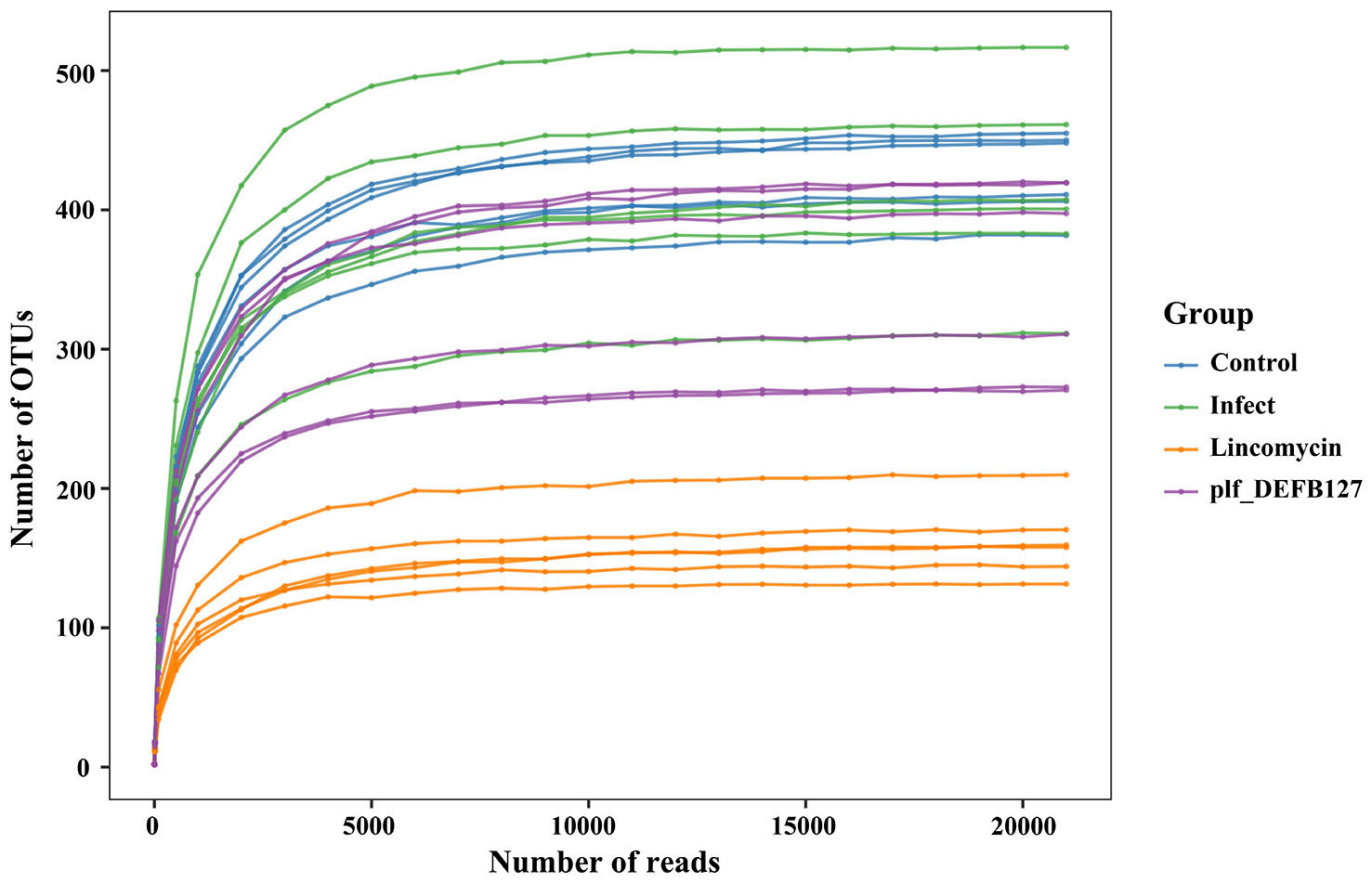
Rarefaction curves (dilution curves) evaluating sequencing depth and sample coverage.

**Figure 11:**
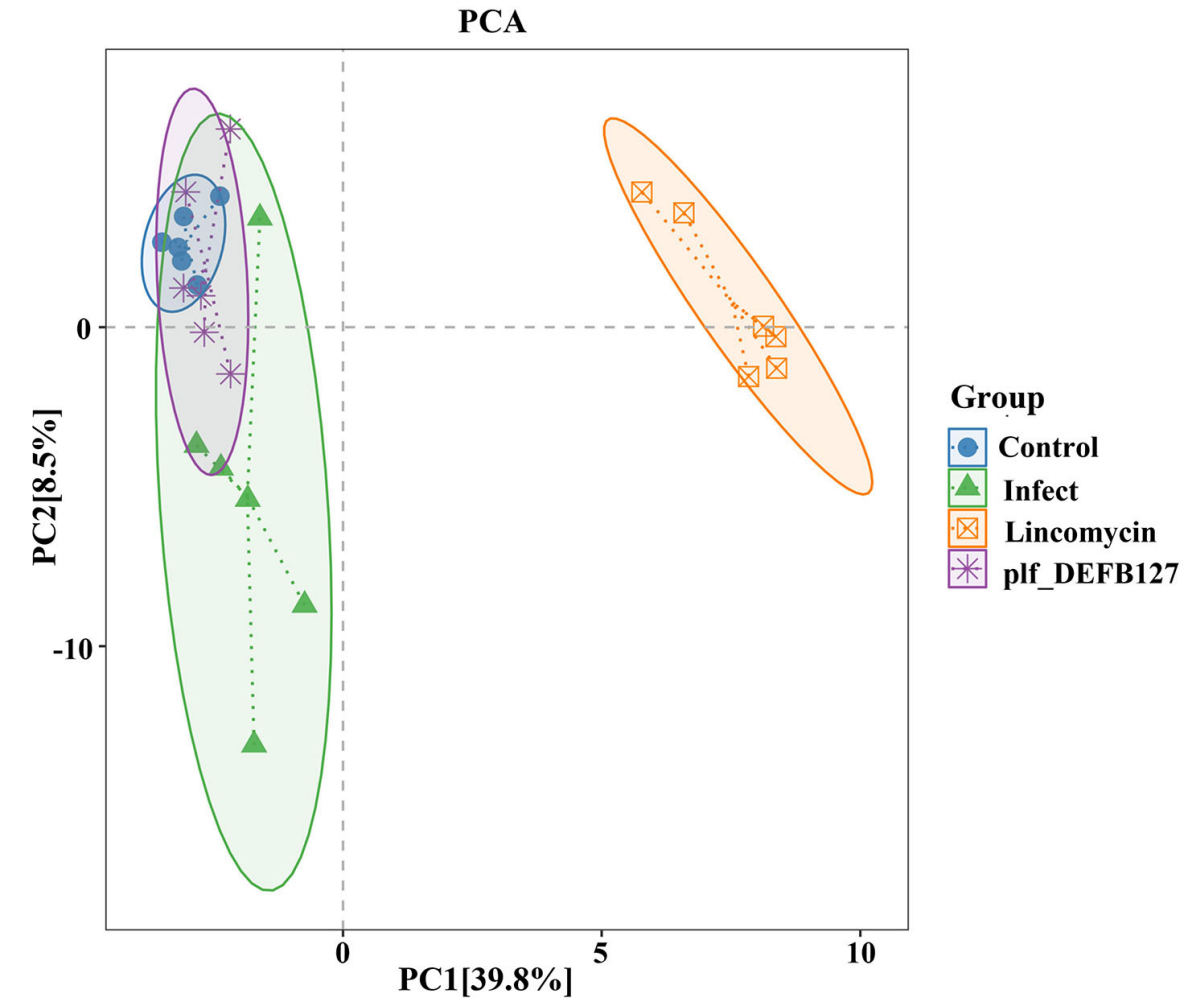
Principal Component Analysis (PCA) plot based on Bray-Curtis dissimilarity at the OTU level.

As shown in Figure 12, a cladogram illustrated the hierarchical taxonomic relationships and relative abundances across all groups. Bacteroidetes was the most abundant phylum, followed by Firmicutes and Proteobacteria, with enriched taxonomic groups including Bacteroidetes (phylum), Bacillales, and Clostridiales (order). Compared with the *S. aureus* and PC groups, the pLF-*DEFB127* group exhibited decreased Proteobacteria and increased Bacillales relative abundance. These microbiota alterations represent associations rather than causal relationships; whether they directly contribute to mastitis alleviation or merely reflect secondary effects of reduced inflammation requires mechanistic investigation. Collectively, pLF-*DEFB127* treatment preserved intestinal microbial diversity and community structure comparable to the NC group, whereas lincomycin induced significant dysbiosis. It is important to note that these microbiota alterations represent associations observed following pLF-*DEFB127* treatment.

**Figure 12:**
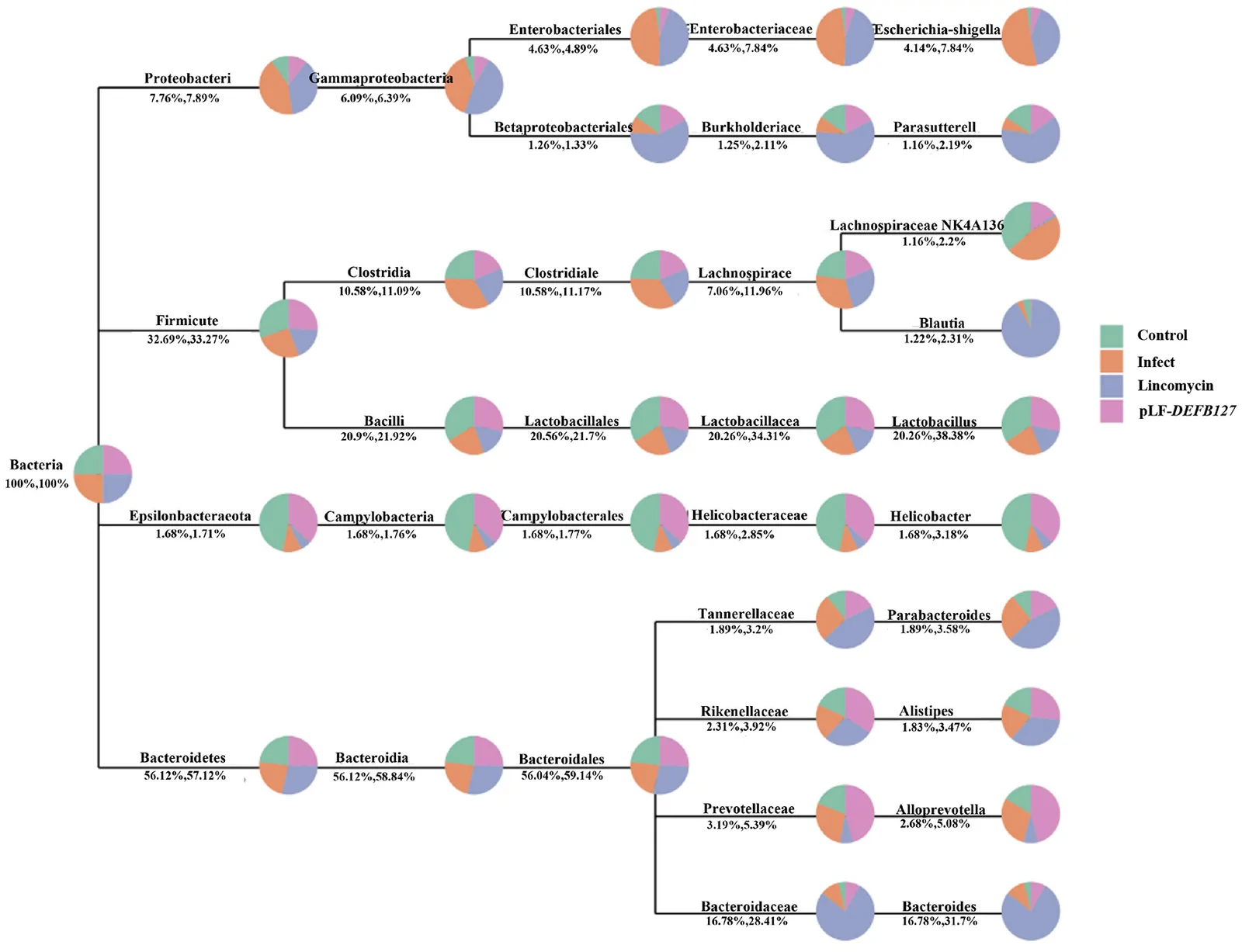
Bacterial taxonomic phylogenetic cladogram. Pie charts at each node show the relative taxon abundance among the four experimental groups (Control, Infect, Lincomycin, pLF–*DEFB127*).

### 3.5 Differences in gut microbiota and correlative prediction of microbiota function

To further characterize the ecological impact of *DEFB127* treatment, α-diversity analysis, differential abundance analysis, and functional prediction were performed. Species diversity was assessed using the Shannon index (Figure 13). The *S. aureus* group exhibited high diversity with considerable inter-individual variability. The PC group showed significantly reduced diversity and low dispersion, indicative of a simplified, homogeneous community structure characteristic of antibiotic-induced dysbiosis. The pLF-*DEFB127* group maintained high diversity comparable to the NC group, though slightly lower, suggesting a stable community structure. Notably, pLF-*DEFB127* treatment preserved microbial community diversity more effectively than conventional antibiotic therapy, as evidenced by significantly higher Shannon diversity relative to the PC group.

**Figure 13:**
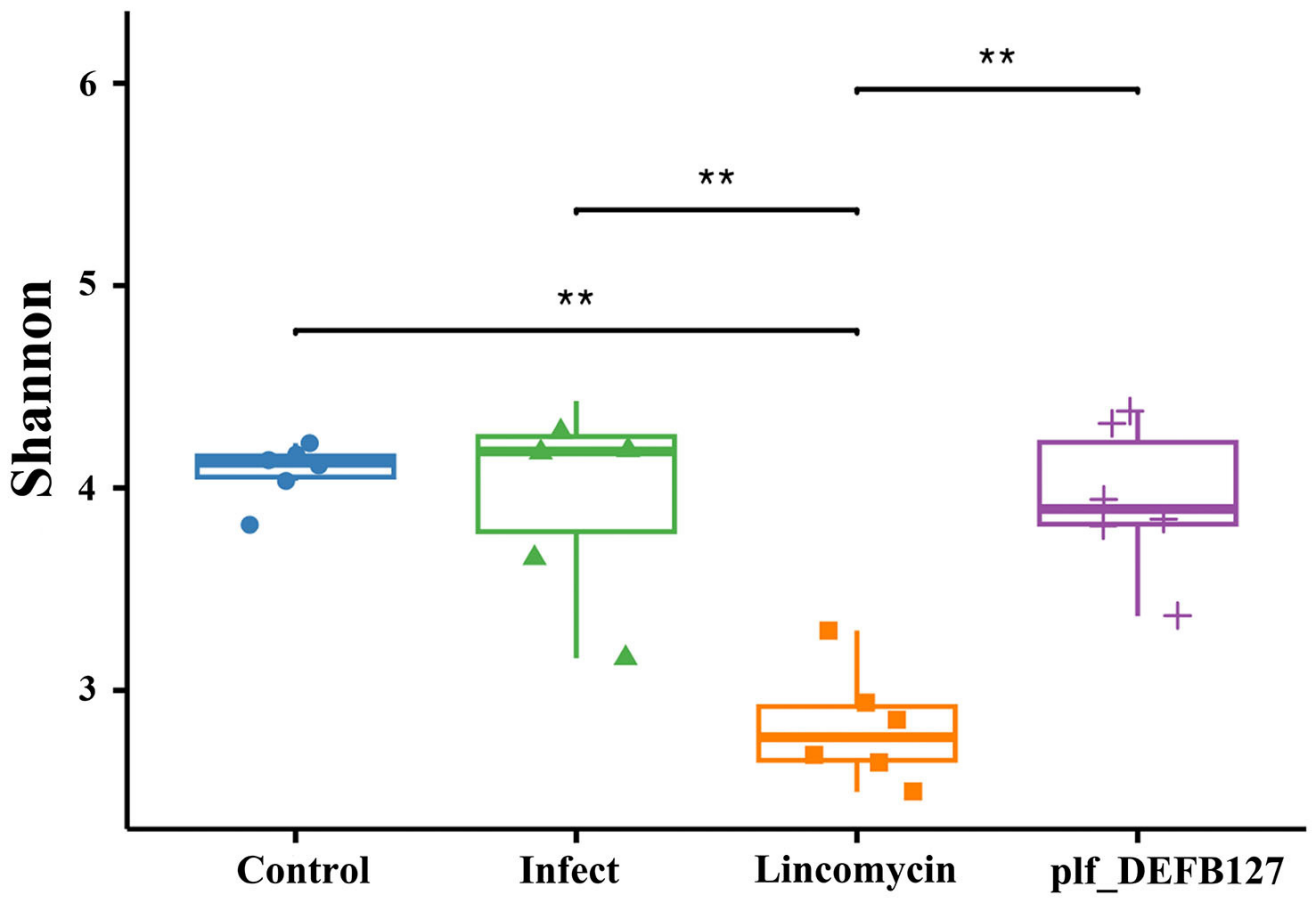
Shannon diversity index. Each dot represents an individual sample, with horizontal lines indicating group means.

LEfSe analysis was performed to identify taxa significantly enriched in specific groups (Figures 14A, B). The taxonomic cladogram (Figure 14B) illustrates discriminatory taxa, with colored nodes representing group-specific enrichment (red, PC; green, pLF-*DEFB127*; blue, NC; yellow, *S. aureus*). LDA scores are presented in Figure 14B. Key findings included: (i) PC group enrichment of Bacteroidaceae (family) and *Bacteroides* (genus); (ii) pLF-*DEFB127* group enrichment of Prevotellaceae (family), Erysipelotrichia (class), Erysipelotrichales (order), and Erysipelotrichaceae (family); (iii) NC group enrichment of *Lactobacillus*-associated taxa (Lactobacillaceae, Lactobacillus, Lactobacillales), Muribaculaceae (family), Bacilli (class), Campylobacterales (order), and Epsilonbacteraeota (phylum); and (iv) *S. aureus* group enrichment of Enterobacteriales (order), Enterobacteriaceae (family), and *Escherichia-Shigella* (genus). These results were consistent with the phylogenetic tree analysis (Figure 12).

**Figure 14:**
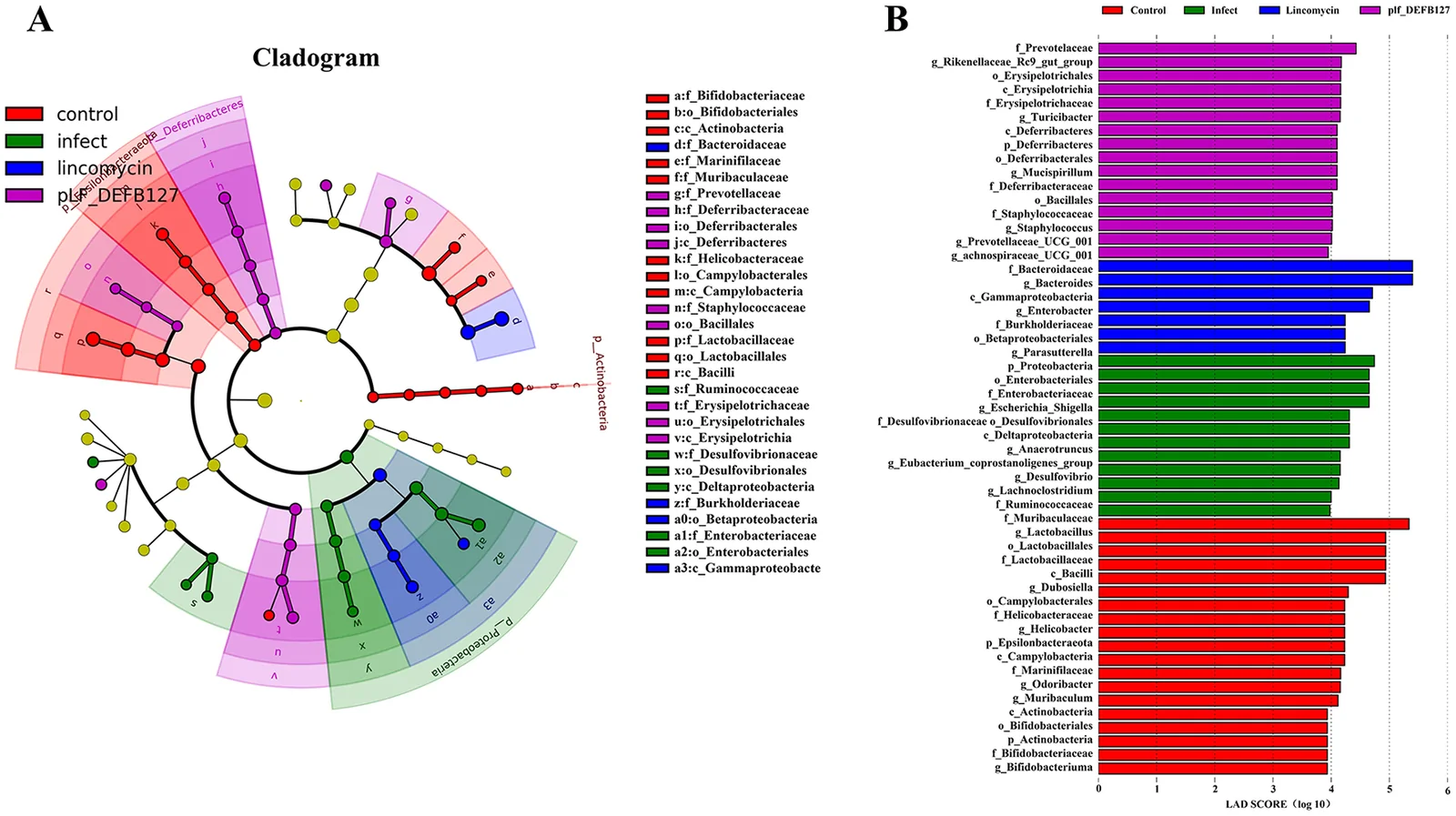
LEfSe analysis of differentially enriched taxa across treatment groups. **(A)** LEfSe taxonomic cladogram displaying significantly enriched taxa in each group. Node size reflects relative abundance; colored nodes indicate statistically significant enrichment (LDA > 2.0, *P* < 0.05). **(B)** Linear Discriminant Analysis (LDA) score plot showing the top discriminatory taxa with LDA scores > 2.0. Positive and negative scores indicate enrichment in the respective groups.

PICRUSt2-based functional prediction of microbial communities from 16S rRNA gene sequences is presented in Figure 15. The stacked bar chart illustrates predicted functional pathway abundance across treatment groups. Notably different functional categories included zeatin biosynthesis, N-glycan biosynthesis, sphingolipid biosynthesis (globo, isoglobo, and ganglioside series), and various host-related pathways (primary immunodeficiency, central carbon metabolism in cancer, anticancer drug resistance, and adipokine signaling), as well as microbial biosynthetic pathways (acarbose and valine biosynthesis). These predictions represent computational inferences from genomic databases and should be considered hypotheses pending experimental validation. Collectively, pLF-*DEFB127* treatment preserved microbial diversity, enriched specific discriminatory taxa, and induced predicted functional alterations, consistent with a microbiota-sparing safety profile relative to conventional antibiotics.

**Figure 15:**
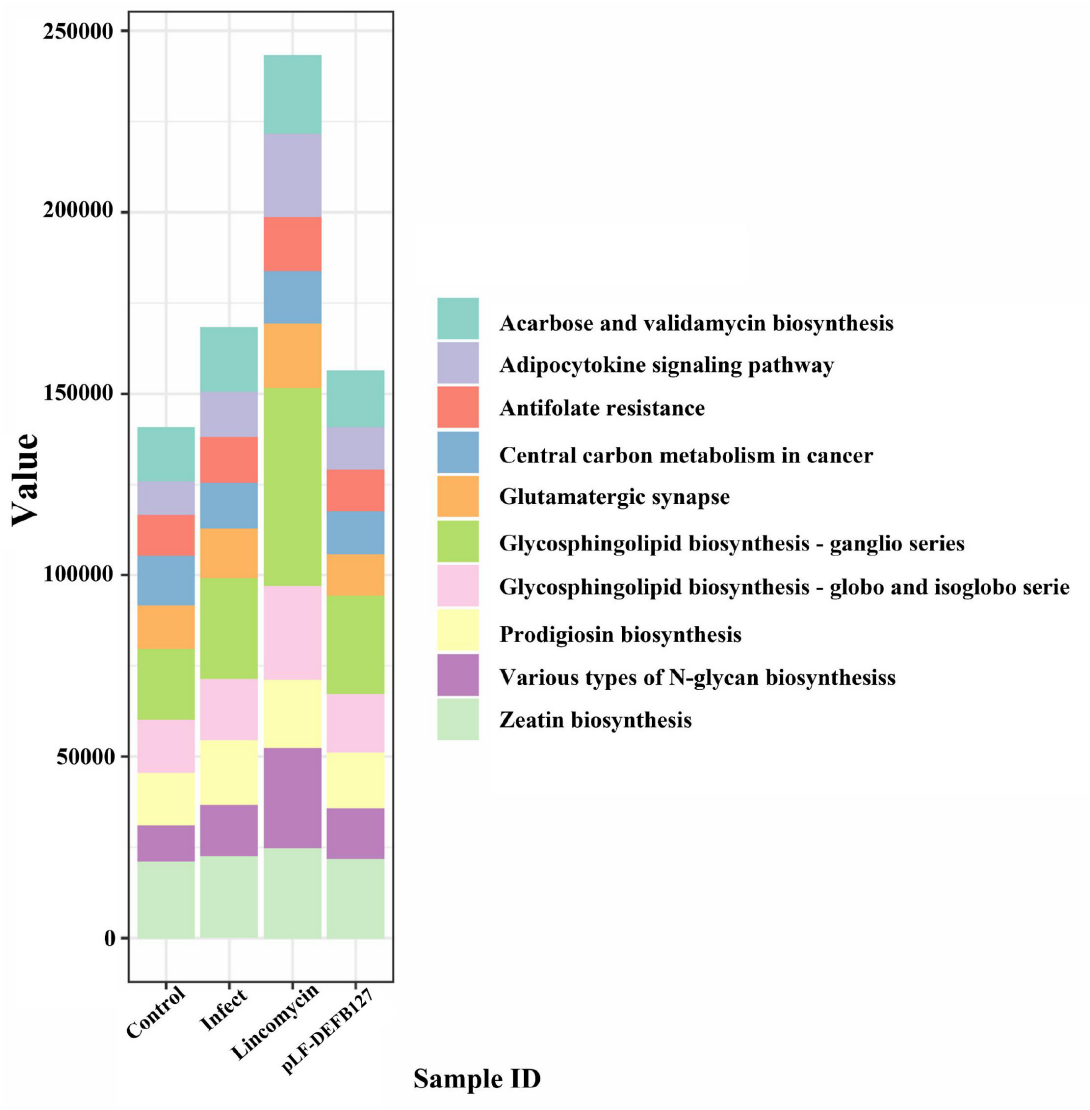
PICRUSt2 functional prediction. Stacked bar chart showing the normalized abundance of predicted KEGG pathways across experimental groups. Bar height represents the total normalized abundance, and each colored segment corresponds to one KEGG functional pathway.

## 4 Discussion

This study addresses whether plasmid-mediated *in situ* expression of yak *DEFB127* can serve as a safe and effective alternative to antibiotics for treating *S. aureus*-induced mastitis. Although *DEFB127* is predicted to possess antimicrobial properties characteristic of β-defensins based on structural analysis, our experimental data demonstrate that its therapeutic efficacy in this model is primarily mediated by anti-inflammatory mechanisms. Our findings support this hypothesis at three levels. First, bioinformatics analysis revealed that yak *DEFB127* possesses the molecular characteristics of functional AMPs, including a high positive charge (+18) and a hydrophobic residue content of 33%, which underpin its membrane-targeting antibacterial mechanism. Second, in both MAC-T cells and a mouse mastitis model, pLF-*DEFB127* significantly downregulated inflammatory cytokines (*TNF-*α, *IL-6*, and *IL-1*β) and ameliorated pathological damage, demonstrating therapeutic efficacy comparable to that of the antibiotic lincomycin. Third, and most notably, 16S rRNA sequencing revealed that pLF-*DEFB127* caused markedly less perturbation to the intestinal microbiota than did lincomycin. Specifically, lincomycin drastically reduced microbial diversity (Shannon index) and altered community structure (PCA), whereas pLF-*DEFB127* maintained a more stable and diverse microbiota profile characterized by increased *Bacillales* and reduced *Proteobacteria*. Collectively, these results establish plasmid-mediated *in situ* expression of yak *DEFB127* as a promising antibiotic alternative that achieves effective mastitis treatment while preserving intestinal microecological homeostasis. However, it should be emphasized that the observed microbiota differences are correlational in nature; whether these changes actively contribute to mastitis recovery or are a passive consequence of improved mammary health remains to be experimentally established.

Due to the negative charge of bacterial cell membranes, cationic AMPs can selectively bind to them, whereas the outer layer of mammalian cell membranes is neutral. Once bound to bacterial membranes, AMPs form pores that increase membrane permeability, thereby leading to bacterial death (19). Signal peptide prediction analysis indicated that the *DEFB127* protein contains a signal peptide cleavage site, and its N-terminal sequence includes a strong hydrophobic region. This facilitates the secretion of AMPs into the extracellular space, thereby enabling their functional role. The bioinformatics characteristics of *DEFB127* identified in this study suggest a potential mechanism by which *DEFB127* may disrupt bacterial cell membranes based on its structural homology to known AMPs (e.g., cationic charge and amphipathic structure). However, our experimental data did not directly demonstrate antibacterial activity; rather, the observed therapeutic effects in both *in vivo* and *in vitro* mastitis models were attributed to the anti inflammatory and immunomodulatory properties of *DEFB127*. The precise molecular mechanisms underlying these anti-inflammatory effects remain to be elucidated. Future studies should investigate whether *DEFB127* modulates key inflammatory signaling pathways such as NF-κB, MAPK, or the NLRP3 inflammasome, as well as its effects on immune cell recruitment, bacterial clearance, cellular apoptosis, and oxidative stress responses in mammary tissue. However, in patients with influenza A H7N9, circulating defensin levels (including *DEFB127*) were positively correlated with blood bacterial load, as reported previously (20). Such differences in homology may lead to amino acid sequence variations in key functional domains, such as structural changes in the antibacterial active center or receptor-binding sites, thereby affecting the binding efficiency and antibacterial spectrum of *DEFB127* with bacterial membranes. Furthermore, differences in evolutionary adaptation between species may have enabled yak *DEFB127* to develop regulatory mechanisms against specific pathogens through long-term natural selection, whereas human *DEFB127* may have lost similar functions due to sequence divergence and may even promote bacterial colonization or amplify inflammatory responses through non-specific binding under specific pathological conditions (21).

Based on the observed associations, we hypothesize that a potential safety advantage of *DEFB127* over conventional antibiotics lies in its differential impact on the host intestinal microbiota. In this study, pLF-*DEFB127* treatment was associated with altered relative abundances of *Proteobacteria* (downregulated) and *Bacillales* (upregulated), whereas lincomycin drastically reduced microbial diversity and altered community structure. These observations suggest that *DEFB127*-based therapy may be more compatible with commensal microbiota preservation than conventional antibiotics; however, whether these compositional changes actively contribute to mastitis recovery or merely reflect reduced mammary inflammation remains to be determined. The gut-mammary axis theory posits that intestinal microbiota can regulate distal organs through pathways such as metabolites, immune cell migration, and hormone-like signaling molecules, with the mammary gland, as an immune-sensitive organ, being particularly responsive to such regulation (22, 23). *Proteobacteria*, as the major phylum of Gram-negative bacteria, exhibit an increase in abundance that is generally regarded as a marker of intestinal dysbiosis and is closely associated with opportunistic infections and elevated inflammatory burden. Previous studies using 16S rDNA sequencing have similarly found that *Proteobacteria* are significantly enriched in the intestines of dairy cows with mastitis and exhibit a positive correlation with plasma metabolites such as 3-epiecdysone, alpha-tocotrienol, dodecanedioic acid, 2-amino-2-deoxyisochorismate, and adenosine (24, 25). This metabolite-microbiota covariation pattern suggests that the aberrant proliferation of Proteobacteria may exacerbate systemic inflammatory responses by disrupting the host endocrine system, such as by interfering with mitochondrial energy metabolism (dodecanedioic acid is an intermediate product of long-chain fatty acid metabolism) (26). Adenosine accumulation, which reflects tissue hypoxia and cellular stress states, constitutes the “intestinal driving force” in the progression of mastitis (27, 28). *DEFB127* is hypothesized to exert a selective regulatory effect that might target this pathological link, though this inference is based on correlational microbiota data rather than direct functional evidence. The downregulation of *Proteobacteria* not only directly reduces the leakage of pathogen-associated molecular patterns (PAMPs) such as lipopolysaccharide (LPS) into the bloodstream, thereby lowering the systemic inflammatory burden on mammary tissue, but also blocks the “gut-leak-mastitis” vicious cycle by improving intestinal barrier integrity, potentially through the restoration of α-tocotrienol's antioxidant function (29). It is important to emphasize that the functional interpretations of the gut-mammary axis discussed in this section are based on correlational 16S rRNA sequencing data and PICRUSt2 computational predictions. These do not constitute direct functional evidence, and the proposed mechanisms should be regarded as hypotheses for future experimental validation.

LDA effect size, as a core indicator for identifying significantly different microbiota between groups, reflects not only the statistical enrichment of taxonomic units in specific treatment groups but also the functional weight of these microbiotas in host physiological remodeling. In this study, the high LDA scores of Erysipelotrichaceae (from class to family) and Prevotellaceae are consistent with the hypothesis that *DEFB127* plasmid-based delivery might reconstruct the microecological basis of gut–mammary gland crosstalk; however, whether this reflects a causal regulatory effect or an epiphenomenon of reduced inflammation requires further investigation. Notably, the enrichment of Erysipelotrichaceae may represent a double-edged effect on metabolic remodeling and immune homeostasis. As a core member of Firmicutes, Erysipelotrichaceae exhibited a high LDA score, indicating its significant ecological advantage in response to plasmid-based delivery. Members of this family generally possess glycolytic and lactic acid-producing capacities, enabling them to ferment various carbohydrates into lactic acid. This metabolic trait helps maintain intestinal pH homeostasis under physiological conditions but may induce ruminal acidosis during dysbiosis, thereby triggering a systemic inflammatory cascade (30). Notably, recent studies have revealed that the abundance of Erysipelotrichaceae UCG-004 in the rumen is positively correlated with milk somatic cell count (SCC), and its increase occurs synchronously with concomitant changes in inflammation-related microbiota in feces, directly linking this family to mastitis status (31). Furthermore, as a pioneer fiber-degrading bacterium of the Bacteroidetes phylum, Prevotellaceae exhibited a high LDA score, which typically indicates an expansion of the carbohydrate-active enzyme (CAZyme) repertoire. This family ferments plant fibers into short-chain fatty acids (SCFAs) such as acetate and propionate by secreting complex polysaccharide-degrading enzymes such as xylanases and pectinases, serving as a cornerstone of intestinal energy homeostasis under healthy conditions (32). However, the association between high LDA scores and mastitis improvement revealed in this study appears to challenge traditional understanding. Emerging evidence indicates that excessive enrichment of Prevotellaceae may pose a “functional overload” risk under specific pathological contexts: whereas its rapid fermentative acid-producing capacity can enhance total SCFA levels, it may also cause a drastic decrease in ruminal pH, triggering subacute ruminal acidosis (SARA), which in turn increases LPS translocation by disrupting epithelial tight junction proteins (ZO-1 and occludin) (32). Notably, LEfSe analysis has identified *Prevotella 9* as a characteristic biomarker (LDA > 4) in the milk microbiota of healthy dairy cows, whereas the subclinical mastitis group exhibits enrichment of *Escherichia-Shigella* and *Lactobacillus* (33). This suggests that the “ecological niche” of Prevotellaceae determines its functional properties, serving as SCFA producers in the intestine and as potential pathogen-excluding commensal bacteria in the mammary gland. The high LDA score of Prevotellaceae in this study may result from the selective enrichment of its subpopulations by yak *DEFB127*: certain *Prevotella* strains (e.g., P. copri) possess a unique tryptophan metabolic pathway that enables them to produce indole-3-propionic acid (IPA), which enhances intestinal IL-22 secretion by activating the AhR, thereby potentially strengthening the epithelial barrier and inhibiting the translocation of *S. aureus* to the mammary gland (32, 33). Therefore, although the observed changes in the gut microbiota of mouse models following plasmid-based delivery of yak *DEFB127* are consistent with a role in promoting recovery from mastitis, it remains equally plausible that these changes are secondary to reduced mammary inflammation rather than causally involved in it. These findings further establish the research foundation for yak *DEFB127* as an alternative to antibiotics for treating bovine mastitis

Finally, the strengths of this study lie in the first application of plasmid-mediated expression of yak *DEFB127* in both *in vivo* and *in vitro* models for treating bovine mastitis, which exhibited significant anti-inflammatory therapeutic efficacy. Furthermore, compared with the lincomycin treatment group, yak *DEFB127* resulted in less perturbation to the gut microbiota, holding promise as an alternative to antibiotics for treating bovine mastitis. The limitations of this study include the lack of in-depth elucidation of the specific mechanisms underlying the therapeutic effect of yak *DEFB127* on bovine mastitis. Specifically, the present study did not investigate the molecular signaling pathways (e.g., NF-κB, MAPK, or the NLRP3 inflammasome) potentially involved in *DEFB127*-mediated anti-inflammatory effects, nor did it assess immune cell infiltration patterns, bacterial clearance efficiency, apoptotic responses, or oxidative stress markers in mammary tissues. A critical limitation is that the gut microbiota analysis presented herein is purely associative; no fecal microbiota transplantation, germ-free mouse experiments, or antibiotic depletion studies were performed to establish whether the observed microbiota alterations are causally linked to mastitis improvement or merely reflect a secondary consequence of reduced mammary inflammation. Furthermore, the functional predictions generated by PICRUSt2 are computational inferences based on 16S rRNA gene sequences and genomic databases rather than direct measurements of microbial metabolic activity or gene expression. Consequently, the proposed functional pathways (e.g., glycosphingolipid biosynthesis and immune signaling) should not be interpreted as experimentally validated biological mechanisms. Consequently, all interpretations regarding the gut-mammary axis in this study should be considered hypotheses requiring experimental validation rather than established conclusions. Furthermore, the absence of experimental correlation between the observed gut microbiota differences and the therapeutic outcomes of bovine mastitis limits the mechanistic conclusions that can be drawn. Further molecular mechanism experiments are still required to validate the results obtained in this study. Second, the present study evaluated *DEFB127* expression exclusively at the transcriptional level (RT-qPCR) and did not validate protein expression, abundance, or localization through Western blotting, immunofluorescence, or immunohistochemistry. Consequently, although plasmid delivery significantly upregulated *DEFB127* mRNA in mammary tissue, this does not necessarily indicate increased production of functional *DEFB127* peptide, which is the biologically active form responsible for the antimicrobial and anti-inflammatory activities of β-defensins. The authors acknowledge that the absence of protein-level validation represents a significant methodological limitation, and the observed therapeutic effects, while associated with *DEFB127* mRNA induction, cannot be definitively attributed to the direct action of functional DEFB127 protein. Future studies incorporating concurrent protein detection assays are essential to establish the causal relationship between DEFB127 protein expression and the observed disease improvement. Future studies will prioritize parallel sample collection to enable comprehensive protein-level validation of *DEFB127* expression and subcellular localization.

## 5 Conclusion

In conclusion, this study demonstrates that plasmid-mediated *in situ* delivery of yak *DEFB127* represents a promising alternative to antibiotics for treating *S. aureus*-induced mastitis through anti-inflammatory mechanisms. Plasmid-based delivery of yak *DEFB127* significantly reduced mammary inflammation and tissue damage, achieving therapeutic outcomes comparable to those of conventional antibiotics. Crucially, unlike lincomycin treatment, pLF-*DEFB127* was associated with minimal perturbation of the intestinal microbiota, characterized by the preservation of microbial diversity and relative enrichment of taxa such as Bacillales. While the causal contribution of these microbiota changes to mastitis recovery remains to be determined, the microbiota-sparing profile of pLF-*DEFB127* represents a promising safety advantage over conventional antibiotics. It is important to acknowledge that, although plasmid delivery resulted in significant upregulation of *DEFB127* mRNA in mammary tissue, protein-level validation of *DEFB127* expression was not performed; therefore, the observed therapeutic effects are associated with *DEFB127* mRNA induction, but the direct contribution of functional *DEFB127* peptide production to disease improvement cannot be definitively established. These findings nonetheless provide preliminary evidence supporting a novel, microbiota-friendly biological strategy for bovine mastitis treatment, offering a viable path toward reducing antibiotic dependence in dairy production.

## Data Availability

The data presented in the study are deposited in the NCBI repository, accession number XM_005887947.1.
